# A multicenter survey of patients’ favorite type of nursing care and associated factors in Hebei Province, China

**DOI:** 10.1371/journal.pone.0264169

**Published:** 2022-03-09

**Authors:** Hongzhi Lv, Yi Cui, Chao Li, Xiaoli Yan, Na Yang, Yadong Wang, Yingze Zhang

**Affiliations:** 1 Editorial Department, The Third Hospital of Hebei Medical University, Shijiazhuang, P.R. China; 2 Department of Orthopedic Surgery, The Third Hospital of Hebei Medical University, Shijiazhuang, P.R. China; 3 Nursing Department, The Third Hospital of Hebei Medical University, Shijiazhuang, P.R. China; Royal College of Surgeons in Ireland, IRELAND

## Abstract

**Background:**

Nursing care service is an important part of the healthcare system; however, patients’ favorite type of nursing care remains unknown. This study aims to investigate inpatients’ and nurses’ favorite types of nursing care and identify nurses’ learning needs.

**Method:**

The study selected a province-representative sample of inpatients and nurses using a stratified random sampling method from 18 selected hospitals, including 9 Level Ⅱ hospitals and 9 Level Ⅲ hospitals in 9 cities of Hebei province. All participants were personally interviewed about their favorite type of nursing care. Multinomial logistic regression analysis was applied to analyze the potential associations between favorite nursing care and factors about inpatients and nurses.

**Results:**

Data from 3,642 inpatients and 371 nurses were included for the final analysis during this survey. Among inpatients, the proportions who selected good attitude-centered, good nursing skill-centered, good environment-centered and good health education guidance-centered as their favorite types of nursing care were 49.9%, 31.8%, 5.8%, and 12.5%, respectively. Concurrently, among nurses, the proportions were 49.9%, 29.6%, 19.1%, and 1.3%, respectively. Multivariate analysis showed that most patients selected good attitude-centered nursing care as their favorite type of nursing care. However, patients who did not live with guardians and had more than one hospitalization per year were more likely to select other nursing care types.

**Conclusion:**

Attitude-centered nursing care service was the favorite type of nursing care for most inpatients and nurses. Health education guidance was another main concern of inpatients. The main factors affecting the patients’ choice of favorite nursing care included patients’ living status and the number of hospitalization events per year. Nursing education should focus on nurse attitude as well as on health education guidance.

## Introduction

Nursing care service is an important part of patient care and improves a patient’s satisfaction with received healthcare [1]. Patient satisfaction is an indicator of the quality of healthcare. A high level of patient satisfaction is closely related to nurses’ satisfaction with their work [2–4]. A high satisfaction rate can also help reduce complaints about healthcare providers and avoid potential medical disputes [5].

In recent decades, patients have become increasingly knowledgeable about healthcare and have increasing demands on healthcare services. Patient-centered nursing care has been recommended and conducted in clinics. The key feature of this care model was the identification of patient needs, which could be analyzed following data collection. However, most nursing care research was conducted from nurses’ point of view, rather than from patients’ perspective, such as the standard and content of nursing services and the design of patient satisfaction questionnaires [6–9]. This study investigated patients’ favorite types of nursing care services, identified nurses’ learning needs, and explored the main factors influencing inpatients’ satisfaction with different nursing care services. We hope this study can provide detailed data to improve nursing care services and the quality of nursing training, thereby increasing inpatients’ satisfaction levels.

## Methods

### Ethical consideration

This study was designed to provide comprehensive information about patients’ favorite types of nursing care services. The Institutional Review Board of The Third Hospital of Hebei Medical University approved the study protocol and written informed consent was obtained from each participant prior to data collection. This study is registered with the Chinese Clinical Trial Registry, number ChiCTR-OOC-1500611.

### Sampling method and study area

**Fig 1:**
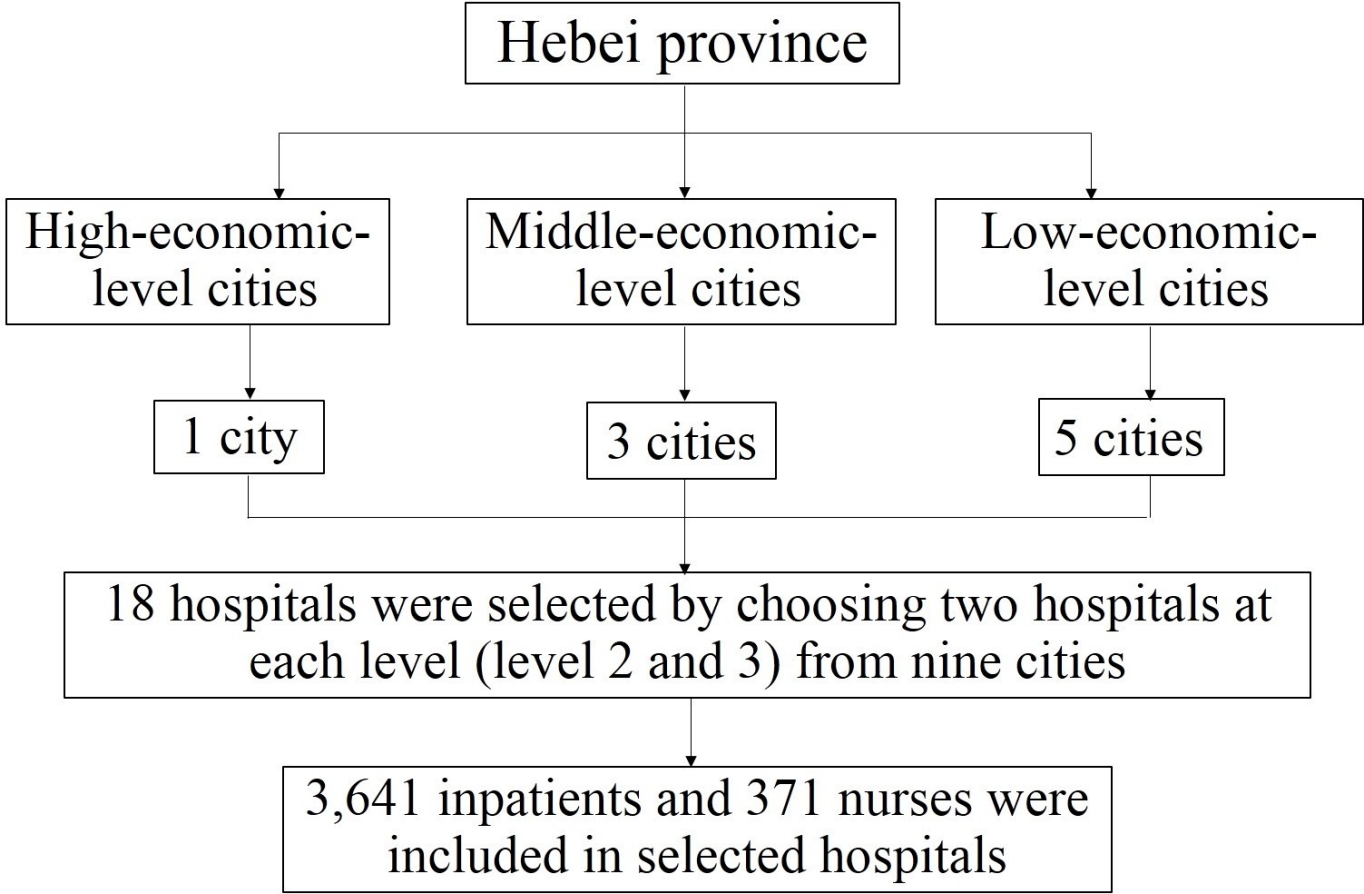
Heibei province study profile of patients’ favorite type of nursing care.

A pilot phase of the study was undertaken at three secondary and three tertiary hospitals in Hebei Province. In total, 1,437 individuals were recruited to estimate the general proportions of patients preferring different types of nursing care services and to facilitate accurate estimation of the sample size needed for the main study from March 2020 to May 2020. The formal study was conducted using stratified cluster random sampling [10, 11]. The 11 cities of Hebei Province were categorized into three economic levels by gross domestic product (GDP); resulting in two high-economic-level cities (GDP > 500 million), Shijiazhuang and Tangshan; three moderate economic level cities (300 > GDP > 500 million), Cangzhou, Handan and Baoding, and six low-economic level-cities (GDP < 300 million), Qinhuangdao, Xingtai, Hengshui, Langfang, Zhangjiakou and Chengde. All hospitals with an inpatient department in Hebei Province were categorized into three levels (primary, secondary and tertiary). Finally, 18 hospitals, including: The Third Hospital of Hebei Medical University, Jingxing Country Hospital, Hengshui people’s Hospital, Jingxian People’s Hospital, Cangzhou Hospital of Integrated TCM-WM, Hejian People’s Hospital, Chengde Central Hospital, Weichang Manchu and Mongolian Autonomous County Hospital, The First Hospital of Qinhuangdao, Lulong County People’s Hospital, The First Central Hospital of Baoding, Dingzhou Maternal and Child Health Care Hospital, Xingtai Mining Group General Hospital, Xingtai People’s Hospital, General Hospital of Jizhong Energy Fengfeng Group, Weixian People’s Hospital, Zhangjiakou Chinese Medicine Hospital, The First Hospital of Zhangjiakou, were selected by choosing two hospitals at each level (level 2 and 3) from nine cities (Shijiazhuang, Hengshui, Cangzhou, Chengde, Qinhuangdao, Baoding, Xingtai, Handan and Zhangjiakou) (Fig 1).

### Study participants

After the hospitals were selected, the patients were first screened using inclusion and exclusion criteria. The inclusion criteria were patients with a hospital stay of greater than 3 days, a stable condition, consciousness, and answering questions clearly. For children in junior and senior high schools, their information was provided by themselves. The exclusion criteria included patients with a history of mental illness, non-cooperative patients, patients incapable of communicating, and critically ill patients. Each patient was assigned a random number.

Using a random number table, 220 patients were randomly selected from the inpatient wards in each hospital, with 5 to 50 patients selected from each department. All the selected patients were personally interviewed, and trained research teams at each hospital completed a questionnaire (S1 and S2 Files). For 1–13 years old children, information was provided by their guardians after asking the patient’s opinion or observing the patient’s performance. If a randomly selected patient refused to participate in the survey, another patient was randomly selected from the same department [12, 13]. The nurses in all the inpatient wards of each department were also interviewed.

### Design of the questionnaire and research team

The questionnaire (S1 and S2 Files) used in this study was designed by our team using the theory of comfort care. We evaluated nursing care service quality across four elements that nurses could provide for patients: a comfortable environment, physiology, psychology, and social culture. The nurses’ attitude, nursing skills, health education guidance, the ward and hospital environment were important aspects of nursing care services that significantly influenced patients’ satisfaction within the health care system. Four types of nursing care services could then be classified based on these dominant aspects: nurse’ attitude-centered, nursing skill-centered, health education guidance-centered and environment-centered nursing care services. Good attitude-centered care services referred to nurses having a high degree of compassion, caring care, active enthusiasm, friendly expression, speaking softly, patient and meticulous work, answering questions, and not quarreling with the patient. Good nursing skill-centered care referred to the ability to practice, evaluate clinical nursing and first aid, provide scientific nursing, and record accurately to ensure quality care. Good environment-centered nursing care services referred to the nurse’s ability to maintain a hygienic, comfortable, safe and quiet environment in the ward, which was suitable for patients to recover their physical and mental health. Good health education guidance-centered care services referred to the nurses providing the patients with concise information about their disease and treatment algorithm. These included exhaustive and useful information on how to prevent inpatients’ complications such as decubitus, deep vein thrombus and hypostatic pneumonia, how to prevent falls and fall-related fractures, and how to act in concert with treatment, professional advice on pain management, rehabilitative training and diet, and concise information about hospital living facilities.

Delphi method was used to analyze the validity of the questionnaire. The draft questionnaire was reviewed by six clinical and nursing specialists and then was modified. It included two rounds of surveys. The first round was an open questionnaire in which experts answered two questions: (1) What do you think a nurse should do to be your favorite type of nursing care and meet your demands? (2) Please describe a particular experience of receiving nursing care and your evaluation of this care. According to experts’ opinions and the results of the first round of investigation, the second round of questionnaires included the above-mentioned four elements that nurses could provide for patients. The authority rating of experts was 0.87±0.09. The expert positive coefficient of both surveys was 100%.

The research team consisted of two physicians, two nursing directors, two head nurses, one epidemiologist, and twenty-seven investigators (including six postgraduate students and 14 nurses). All the team members undertook a 1-week, centralized training program and then were stratified into six subgroups. A questionnaire was administered by trained staff to obtain information about the inpatients’ demographic characteristics, personal medical status, demand for nursing care services, and basic information about the nurses.

The questionnaire used to interview patients included: age, occupation, gender, residence, living status, education level, inpatient department, times of admission, number of hospitalization per year, working status. All the selected inpatients answered the question above and their favorite type of nursing care. The respondents were classified into two groups: children younger than 14 years old, whose questionnaires were completed following interviews with their parents or other adults after asking the patient’s opinion or observing the patient’s performance; and adults and children over 14 years old were interviewed personally [12]. The inpatients’ medical records were reviewed. The data were extracted included: health insurance, postoperation complication, adverse events in the hospital, nursing classification, preoperative comorbidities, length of hospital stays, hospital level, extra bed ward and Modified Early Warning Score (MEWS). The questionnaire related to nurses included age, gender, education level, nurse-staffing levels, and years of working as a nurse. All selected nurses answered the most important aspect of being a nurse, which was recorded as nurses’ favorite types of nursing care to make the expression consistent with the questionnaire of patients.

### Data collection and entry and quality control

Nine quality control teams were established (one per city), and they sampled 10% of all the questionnaires collected in the field to check for omissions or errors. All the data were recorded on a written survey at each household selected and were later entered into the EpiData 3.1 software program using the dual import program. The dually imported data were then compared, and any mismatched information was corrected using the original version.

### Statistical analysis

All statistical analyses used SPSS 13.0 (IBM, Armonk, NY, USA). Multinomial logistic regression models were used to assess the relationship between the four types of nursing care and various factors of inpatients, including: age, occupation, gender, residence, living status, education level, inpatient department, times of admission, number of hospitalization per year, health insurance, postoperation complication, adverse events in hospital, nursing classification, preoperative comorbidities, MEWS score, length of hospital stays, working status, hospital level and extra bed ward. The potential correlations between the four types of nursing care and various factors of nurses, including: age, gender, education level, nurse-staffing levels, and years working as a nurse, were also studied by multinomial logistic regression models using the four types of nurses as the reference category. A stepwise strategy was used to select the confounding factors. Adjusted odds ratios (ORs) with 95% confidence intervals (CIs) were calculated for all the models. Pearson’s *χ*^*2*^ test was used to compare the difference between the nurses’ and patients’ opinions about the ‘patients’ favorite nurse’. A *P* value < 0.05 (two-tailed) was considered statistically significant.

## Results

The pilot phase of the survey showed that 3.7% of the questionnaires were unusable; thus, 5% was added to the total required sample size for the main study. In total, 3,780 patients were selected and invited to participate in the study, among whom 139 (3.7%) were ultimately excluded because of missing items, lack of response, or logical errors in the questionnaire. The remaining 3,641 patients (96.3%), among whom 1,781 (48.9%) were male and 1,860 (51.1%) female, were enrolled for the final analysis (Table 1).

**Table 1:** Basic information concerning respondents’ demographic characteristics.

Variables	Keep a good attitude	Good nursing technology	Good environment	Good health education guidance	*χ*^*2*^ value	*P* value
Gender						
Male	103	186	588	904	13.640	0.003*
Female	109	268	569	914		
Occupation						
Student/Preschool children	30	49	172	170	57.428	<0.01*
Manual worker	26	45	117	221		
Farmer	103	203	539	859		
Retired	21	51	156	266		
Civil servant	6	29	60	80		
Medical personnel	4	4	5	12		
Military personnel	1	0	3	3		
Others^a^	21	73	105	207		
Age(years)						
0–10	24	41	143	123	70.965	<0.01*
11–20	7	9	34	50		
21–30	32	78	105	201		
31–40	18	61	86	153		
41–50	23	46	131	212		
51–60	34	76	214	334		
61–70	39	87	228	400		
71–80	22	43	150	236		
80+	13	13	66	109		
Residence						
Rural	63	153	406	626	2.384	0.497
Urban	149	301	751	1192		
Living status						
Single	15	23	59	88	34.127	0.001*
with spouse	71	148	368	583		
With children	16	45	127	222		
With children and spouse	73	167	407	726		
With guardians	37	71	196	199		
Education level						
Elementary school graduate or less	77	138	469	653	24.166	0.004*
Middle school graduate	58	125	303	489		
High school graduate	50	102	244	399		
College graduate or higher	27	89	141	277		
Inpatient department						
Surgical department	83	173	412	757	100.783	<0.01*
Internal medicine	78	137	490	732		
Gynecology-obstetrics	27	103	108	201		
Pediatrics	19	38	133	117		
Others	5	3	14	11		
Times of admission						
1–5	208	441	1113	1771	4.933	0.552
6–10	3	11	36	37		
10+	1	2	8	10		
Number hospitalization per year						
<once	151	310	799	1369	23.653	0.001*
= once	36	96	237	322		
>once	25	48	121	127		
Health insurance						
Non	30	35	114	177	13.309	0.347
Medical insurance for urban workers/residents	37	95	248	393		
New rural cooperative medical system	140	315	751	1189		
Commercial health insurance	2	2	12	14		
Free medical service	3	7	32	45		
Postoperation complication						
Non	204	446	1136	1780	3.638	0.303
Yes	8	8	21	38		
Adverse events in hospital						
Non	211	454	1154	1817	4.506	0.212
Yes	1	0	3	1		
Nursing classification						
grade three care	14	25	40	56	19.570	0.021*
grade two care	160	376	949	1534		
grade one care	32	45	141	196		
special level care	6	8	27	32		
Preoperative comorbidities						
Non	174	357	852	1294	19.207	<0.01*
Yes	38	97	305	524		
MEWS score						
<1	61	122	233	347	35.654	<0.01*
= 1	111	250	680	1125		
= 2	21	48	126	224		
>2	19	34	118	122		
Length of hospital stays (days)						
3–7	145	316	760	1123	20.703	0.055
8–14	46	88	253	399		
15–21	11	23	74	143		
22–28	4	10	24	55		
28+	6	17	46	98		
Working status						
Inservice	40	89	161	321	33.746	0.001*
Farming	92	181	492	781		
Preschool children	20	32	118	106		
Students	9	17	51	61		
Retirement	51	135	335	549		
Hospital level					49.953	<0.01*
= 1	401	368	64	128		
= 2	649	313	58	123		
= 3	768	476	90	203		
Extra bed ward						
Non	148	330	849	1298	2.018	0.569
Yes	64	124	308	520		

^a^ Others mean freelancers and unemployed personnel.

*means *P*<0.05

The general characteristics and health status of the 3,641 selected patients are shown in Table 1. The largest groups of patients were those aged between 51 and 70 years old (38.8%) and farmers (46.9%). The number of male and female patients was similar (48.9% and 51.1%). There were more patients from urban areas (65.7%) than rural areas (34.3%). The proportions of the inpatients who selected good attitude-centered nursing care, good nursing skill-centered care, good environment-centered nursing care and good health education guidance-centered nursing care as their favorite types of nursing care were 49.9%, 31.8%, 5.8% and 12.5%, respectively.

The basic characteristics of the surveyed patients’ nurses and departments are shown in Table 2. The average number of beds in each department was 41.5 ± 10.8 (range 4–87), and the average number of inpatients in each department was 37.1 ± 13.0 (range 1–92). The average number of nurses in each department was 12.1 ± 4.3 (range 1–37), and the average number of day-shift nurses was 4.3 ± 2.1 (range 1–16).

**Table 2:** Basic information of department and nurses in charge with patients surveyed.

Variables	Keep a good attitude	Good nursing technology	Good environment	Good health education guidance	*χ*^*2*^ value	*P* value
Nurses’ age(years)						
18–30	128	275	688	1064	10.096	0.343
31–40	72	154	373	591		
41–50	11	24	85	152		
50+	1	1	11	11		
Nurses’ gender						
Male	1	3	5	2	5.019	0.170*
Female	211	451	1152	1816		
Nurses’ education level						
Junior college or less	7	9	19	33	16.260	0.062
bachelor	123	249	714	1019		
Master	82	193	421	761		
Doctor	0	3	3	5		
Nurse-staffing levels						
Registered nurse	39	82	183	286	10.002	0.616
Nurse practitioner	135	284	732	1144		
Supervisor nurse	37	82	232	354		
Co-chief nurse	1	5	9	28		
Chief nurse	0	1	1	6		
Years worked as a nurse						
1–10	164	336	827	1274	16.548	0.056
11–20	34	99	234	417		
21–30	13	19	92	123		
31–40	1	0	4	4		

^a^ Others mean freelancers and unemployed personnel.

*means *P*<0.05

In total, 371 nurses participated in the survey, among whom 362 were female (97.6%), and 9 were males (2.4%). Additionally, 11 participants were registered nurses (3.0%), 73 were nurse practitioners (19.7%), 223 were charge nurses (60.1%), 53 were co-chief superintendent nurses (14.3%), and 11 were superintendent nurses (3.0%). The proportions of the nurses who selected good attitude-centered nursing care, good nursing skill-centered care, good environment-centered nursing care and good health education guidance-centered nursing care as patients’ favorite types of nursing care were 49.9%, 29.6%, 19.1% and 1.3%, respectively. A statistically significant difference was found between the nurses and inpatients (*χ*^*2*^ = 121.505, *P* < 0.01).

Multinomial logistic regression was performed, with the characteristics of the patients, nurses and departments as covariates to predict patients’ satisfaction with nursing services (Tables 3–6). Most patients selected good attitude-centered nursing care as their favorite type of nursing care (Table 3). However, patients who were cared for by male nurses (*OR*: 7.224; 95% *CI*: 1.125–46.399) and received care in a department with fewer day-shift nurses (OR: 1.112; 95% *CI*: 1.052–1.177) were more likely to select good nursing technology as their favorite type of nursing care (Table 4). The patients who lived with children (*OR*: 0.386; 95% *CI*: 0.192–0.778) or with children and a spouse (*OR*: 0.392; 95% *CI*: 0.210–0.732), single patients (*OR*: 0.462; 95% *CI*: 0.219–0.971), patients with a spouse (*OR*: 0.459; 95% *CI*: 0.247–0.854), and patients with no more than one hospitalization per year (*OR*: 0.493; 95% *CI*: 0.336–0.723) were more likely to select good health education guidance-centered nursing care as their favorite type of nursing care (Table 5). The patients who lived with children (*OR*: 0.256; 95% *CI*: 0.092–0.716) or who had preoperative comorbidities (*OR*: 1.696; 95% *CI*: 1.123–2.562) and those in internal medicine (*OR*: 0.348; 95% *CI*: 0.103–1.177), gynecological-obstetric (*OR*: 0.263; 95% *CI*: 0.071–0.980) or pediatric wards (*OR*: 0.128; 95% *CI*: 0.029–0.557) were more likely to choose good environment-centered nursing care as their favorite type of nursing care (Table 6).

**Table 3:** Risk factors for patients’ favorite nurse with good attitude.

	*B*	*SE*	*Wald*	*P* value	*OR*	95%CI		
Hospital level			48.183	<0.01				
= 1	-0.480	0.112	18.356	<0.01*	0.619	0.497	~	0.771
= 2	0.205	0.100	4.240	0.039*	1.227	1.010	~	1.492
Living status			15.246	0.004				
With spouse	0.115	0.166	0.482	0.488	1.122	0.810	~	1.555
With children	0.384	0.187	4.235	0.040*	1.468	1.018	~	2.117
With children and spouse	0.281	0.166	2.861	0.091	1.325	0.956	~	1.834
With guardians	-0.448	0.265	2.848	0.091	0.639	1.380	~	1.075
Inpatient department			5.438	0.245				
Surgical department	0.682	0.398	2.934	0.087	1.978	0.906	~	4.315
Internal medicine	0.603	0.400	2.267	0.132	1.828	0.834	~	4.007
Gynecology-obstetrics	0.591	0.416	2.022	0.155	1.806	0.800	~	4.079
Pediatrics	1.029	0.477	4.656	0.031*	2.799	1.099	~	7.131
Times of hospitaliztion every year			11.785	0.003				
<once	0.406	0.137	8.773	0.003*	1.501	1.147	~	1.964
= once	0.192	0.148	1.676	0.195	1.211	0.906	~	1.619
Preoperative comorbidities	-0.186	0.088	4.468	0.035*	0.830	0.699	~	0.987
Length of hospital stays (days)			11.886	0.018				
3–7	-0.408	0.173	5.565	0.018*	0.665	0.473	~	0.933
8–14	-0.340	0.180	3.549	0.060	0.712	0.500	~	1.014
15–21	-0.070	0.209	0.113	0.737	0.932	0.619	~	1.404
22–28	-0.019	0.271	0.005	0.943	0.981	0.576	~	1.670
Total nurses number	-0.044	0.011	16.175	<0.01*	0.957	0.937	~	0.978
Day shift duty nurses	-0.043	0.021	4.424	0.035*	0.958	0.920	~	0.997

*means *P*<0.05.

**Table 4:** Risk factors for patients’ favorite nurse with good nursing technology.

	*B*	*SE*	*Wald*	*P* value	*OR*	95%CI
Hospital level			31.095	<0.01		
= 1	0.450	0.117	14.877	<0.01*	1.568	1.247~1.970
= 2	-0.117	0.108	1.172	0.279	0.890	0.721~1.099
MEWS score			5.523	0.137		
<1	-0.330	0.154	4.593	0.032*	0.719	0.532~0.972
= 1	-0.153	0.140	1.202	0.273	0.858	0.652~1.128
= 2	-0.222	0.168	1.732	0.188	0.801	0.576~1.115
Total nurses number	0.036	0.011	11.457	0.001*	1.037	1.015~1.059

*means *P*<0.05.

**Table 5:** Risk factors for patients’ favorite nurse with good health education guidance.

	*B*	*SE*	*Wald*	*P* value	*OR*	95%CI		
Times of hospitaliztion every year			14.130	0.001				
<1	-0.544	0.193	7.920	0.005*	0.580	0.397	~	0.848
= 1	-0.119	0.207	0.333	0.564	0.887	0.591	~	1.332
Number of beds	0.018	0.008	4.614	0.032*	1.018	1.002	~	1.034
Total patients number	-0.022	0.008	7.859	0.005*	1.008	1.003	~	1.009
Day shift duty nurses	0.129	0.029	20.292	<0.01*	1.137	1.075	~	1.203

*means *P*<0.05.

**Table 6:** Risk factors for patients’ favorite nurses with a good environment-centered care.

	*B*	*SE*	*Wald*	*P* value	*OR*	95%CI		
Living status			7.201	0.126				
with spouse	-0.220	0.313	0.494	0.482	0.803	0.435	~	1.481
With children	-0.880	0.390	5.099	0.024*	0.415	0.193	~	0.890
With children and spouse	-0.444	0.315	1.989	0.158	0.641	0.346	~	1.189
With guardians	-0.039	0.478	0.007	0.934	0.961	0.377	~	2.454
Inpatient department			5.472	0.242				
Surgical department	-0.720	0.574	1.571	0.210	0.487	0.158	~	1.500
Internal medicine	-0.681	0.584	1.359	0.244	0.506	0.161	~	1.590
Gynecology-obstetrics	-1.059	0.632	2.813	0.093	0.347	0.101	~	1.195
Pediatrics	-1.372	0.688	3.975	0.046*	0.254	0.066	~	0.977
Extra bed ward	0.435	0.206	4.441	0.035*	1.545	1.031	~	2.315

## Discussion

Patient satisfaction has been widely used worldwide to assess whether the available healthcare supply can meet patients’ health needs and expectations and was valuable to evaluate medical effectiveness, nursing care service and healthcare staff from the patient’s perspective [8, 14–16]. Improving the quality of nursing care services has become an urgent problem [17–19]. To our knowledge, this study is the first to investigate patients’ preferences for care services in China. It used a stratified random sampling method to compare inpatients’ and nurses’ opinions about patients’ favorite types of nursing care services across 18 hospitals. The key feature of this study was identifying patients’ needs through the collection of factual data. Our findings suggested that attitude-centered nursing care services were the favorite type of nursing care for most of the inpatients, a finding that was consistent with the results of several other studies [3, 20, 21]. Notably, the proportion of patients who selected good health education guidance-centered as their favorite type of nursing care was 12.5%, while that among nurses was 1.3%. Since the patients lacked professional knowledge, they needed more professional guidance. As such, this was the main difference between the patients and nurses. Nursing education should focus more on health education guidance in addition to nurse attitude.

Previous studies have reported several factors that influence patient satisfaction with nursing care [22–25]. Barber *et al*. [26] reported that race influenced patient satisfaction. Li *et al*. [27] argued that self-evaluation of health, education level, being Asian or White, and being prescribed a new medication; all affected satisfaction. A study in France reported that the main factors influencing patients’ satisfaction were patient age, quality of life, physician’s concern, interest in the medical problem, and focus on the symptoms [4]. Balaguer *et al*. [28] reported that the efficiency of the physicians and clarity of information were two of the most important factors in overall satisfaction. One study found that patient age and health status were important elements influencing satisfaction [29], although another found no relationship among age, education and patient satisfaction [30]. Statistical analysis of the current study showed that nearly half of the patients preferred good attitude-centered nursing care, followed by good nursing skill-centered nursing care, good health education guidance-centered nursing care, and good environment-centered nursing care. From the nurses’ perspective, the patients’ favorite type of nursing care was good attitude-centered care, followed by good nursing skill-centered care, good environment-centered nursing care, and good health education guidance-centered nursing care. The multinomial logistic regression showed that patients who chose good attitude-centered care as the favorite type of nursing care were more likely to have stayed in a level 2 hospital, had less than one hospitalization per year, and were treated in internal medicine, gynecology-obstetric or pediatric wards. The patients without preoperative comorbidities were more likely to choose good environment-centered care as their favorite type of nursing care. Those who were cared for by male nurses or in a department with fewer day-shift nurses tended to choose good health education guidance-centered care as the favorite type of nursing care. Patients in departments with fewer nurses, in level 1 hospitals, with a MEWS score of more than 2 or who stayed in the hospital for 3–7 days preferred good nursing skill-centered care.

Wiechula adopted Umbrella review methodology to screen out 12 reviews (representing over 290 studies) for evaluation and considered the factors influencing the caring relationship between a nurse and patient. He found that communication, environment, knowledge and skills were the main influencing factors [31]. Evidence from 34 literature reviews suggested that nurses’ professional competencies and attitude were important in developing a trusting relationship [32]. These conclusions were consistent with the results of this study. The increasingly complex requirements of today’s nursing practitioners have been accompanied by demands on nurse educators to improve the situation to facilitate learning in the clinical area [33]. The proportion of schools teaching nurses increased from 20.8% in 1999 to 52.8% in 2014 [34]. However, only a small proportion of nurses in practice have formal nursing education beyond senior high school. This problem exists in the United States but is worse in China because nursing education has been offered for a much shorter period [35]. This study has provided comprehensive information about the important aspects of nursing education to improve the quality of nursing care provision.

To our knowledge, this study is the first multicenter study to investigate the patients’ favorite type of nursing care in China but has some limitations. First, for 1–13 years old children, information was provided by their guardians. The opinions of their guardians could not fully reflect their children’s feelings. Second, for 1–13 years old children, information was provided by their guardians after asking the patient’s opinion. But there could be a mix of guardian preference, especially for children between the ages of one and five. Additionally, the data did not provide information about treatment effectiveness. Future research should focus on country-level analysis, considering more factors and evaluating the use of the questionnaire and possible ceiling effect.

## Conclusion

Attitude-centered nursing care service was the favorite type of nursing care for most of the inpatients and nurses. Health education guidance was another main concern of inpatients. The main factors affecting patients’ choice of nursing care included patients’ living status and hospitalization events per year. Nursing education should focus more on nurse attitude and health education guidance.

### Relevance to clinical practice

Based on our findings, nursing education should focus more on nurses’ attitudes, health education guidance, and improving their communication and general nursing skills.

## Supporting information

S1 File The original language questionnaire in multicenter survey of patients’ favorite type of nursing care. (DOC)

S2 File The English language questionnaire in multicenter survey of patients’ favorite type of nursing care. (DOC)

S3 File The minimal data. (XLSX)

## Abbreviation

MEWS: Modified Early Warning Score
OR: odds ratio
CI: confidence interval
GDP: gross domestic product
