# Supplementary material for: A multicenter survey of patients’ favorite type of nursing care and associated factors in Hebei Province, China
Source: PLoS One. 2022 Mar 9;17(3):e0264169. doi: 10.1371/journal.pone.0264169 (PMC8906638; doi:10.1371/journal.pone.0264169)
Supplement: S1 File — (DOC) [file pone.0264169.s001.doc]

**医院 科室 床号 病案号**

**患者相关表格**

**第一部分 基本资料**

A1 年龄 岁

A2 性别 1=男 2=女 □

A3 文化程度：1=小学毕业及以下 2=初中毕业 3=高中毕业 4=大专及以上学历 □

A4 居住地：1=城市 2=农村 □

A5 职业（当前，包括返聘者当前职业）  □

1=农民 2=学生/学龄前儿童 3=工人 4=军人 5=公务员 7=医务人员 8=退休人员 9=其他（注明 ）

A6 工作状态：1=在职 2=务农 3=退休 4=学龄前儿童 □

A7 入院次数_______次

A8 居住状态：1=独居 2=仅与老伴同住 3=与子女同住 4=与老伴及子女同住

5=和监护人同住 □

A9 每年住院次数_______

**第二部分 住院患者病历资料**

B1 医疗保险（可多选） □□

0=无 1=城镇职工/居民医疗保险 2=新型农村医疗合作制度 3=商业医疗保险 4=公费医疗

B2 护理级别_______? □

1=特级护理 2=Ⅰ级护理 4=Ⅱ级护理 5=Ⅲ级护理

B3 术后并发症 □

0=无 1=有

B4 住院期间的不良反应 □

0=无 1=有

B5 术前合并症 □

0=无 1=有

B6 住院时长_______天

B7 加床病房 □

0=否 1=是

B8 MEWS得分_______?

改良的早期预警评分（MEWS）

| 项目 | 评分 | | | | | | |
| --- | --- | --- | --- | --- | --- | --- | --- |
|  | 3 | 2 | 1 | 0 | 1 | 2 | 3 |
| 心率(次/min) |  | <=40 | 41-50 | 51-100 | 101-110 | 111-129 | >=130 |
| 收缩压(mmHg) | <=70 | 71-80 | 81-100 | 101-199 |  | >=200 |  |
| 呼吸频率(次/min) |  | <9 |  | 9-14 | 15-20 | 21-29 | >=30 |
| 体温(摄氏度) |  | <35 |  | 35.0-38.4 |  | >=38.5 |  |
| 意识 |  |  |  | 清楚 | 对声音有反应 | 对疼痛有反应 | 无反应 |

**第三部分 你最喜欢的护理服务**

C1 你最喜欢哪种护理服务？ □

1=以好的态度为中心的护理服务 2=以好的护理技能为中心的护理服务 3=以好的环境为中心的护理服务 4=以好的健康指导为中心的护理服务

**注：如果您未满14岁，请由您的监护人代为填写**

**患者所在科室和主管护士的基本信息**

A1 年龄 岁

A2 性别 1=男 2=女 □

A3 文化程度

1=大专及以下 2=本科 3=硕士 4=博士 □

A4 职称

1=护士 2=护师 3=主管护师 4=副主任护师 5=主任护师 □

A5 从事护士工作时间 □

1=1-10 2=11-20 3=21-30 4=31-40

B1 作为一名护士，你认为最重要的方面是什么？ □

1=好的工作态度 2=好的护理技术 3=保持良好的环境 4=提供好的健康教育指导

调查人____________ 调查时间_____年______月______日

审核人____________ 审核时间_____年______月______日
