# Supplementary material for: A multicenter survey of patients’ favorite type of nursing care and associated factors in Hebei Province, China
Source: PLoS One. 2022 Mar 9;17(3):e0264169. doi: 10.1371/journal.pone.0264169 (PMC8906638; doi:10.1371/journal.pone.0264169)
Supplement: S2 File — (DOC) [file pone.0264169.s002.doc]

**Department Bed number Medical record number**

**Patient related form**

**Part I Basic information**

A1 Age years

A2 Gender 1=Male 2=Famale □

A3 Education level：1= Elementary school graduate or less 2= Middle school graduate 3= High school graduate 4= College graduate or higher □

A4 Residence：1=Urban 2=Rural □

A5 Occupation (Current, Including rehiring as a current professional) □□

1=Farmer 2=Student/Preschool children 3=Manual worker 4=Military personnel 5=Civil servant 7=Medical personnel 8=Retired 9= Others（give clear indication of ）

A6 Working status: 1=Inservice 2=Farming 3=Retirement 4=Preschool children

5= Students □□

A7 Times of admission times

A8 Living status: 1= Single 2=With spouse 3=With children 4=With children and spouse

5= With guardians □

A9 Number hospitalization per year

**PartⅡ Inpatients’ medical records**

B1 Health insurance（Multiple choices are allowed） □□

0=Non 1=Medical insurance for urban workers/residents 2=New rural cooperative medical system 3=Commercial health insurance 4= Free medical service

B2 Nursing classification ？ □

1=special level care 2= grade one care 3= grade two care 4= grade three care

B3 Postoperation complication □

0=Non 1=Yes

B4 Adverse events in hospital □

0=Non 1=Yes

B5 Preoperative comorbidities □

0=Non 1=Yes

B6 Length of hospital stays days

B7 Extra bed ward □

0=Non 1=Yes

B8 MEWS score ？

Modified Early Warning Score（MEWS）

| Item | Score | | | | | | |
| --- | --- | --- | --- | --- | --- | --- | --- |
|  | 3 | 2 | 1 | 0 | 1 | 2 | 3 |
| HR(times/min) |  | <=40 | 41-50 | 51-100 | 101-110 | 111-129 | >=130 |
| SBP(mmHg) | <=70 | 71-80 | 81-100 | 101-199 |  | >=200 |  |
| R(times/min) |  | <9 |  | 9-14 | 15-20 | 21-29 | >=30 |
| T(℃) |  | <35 |  | 35.0-38.4 |  | >=38.5 |  |
| Consciousness |  |  |  | Clear | Respond to sound | Respond to pain | No reaction |

**PartⅢ Your favorite nursing care service**

C1 Which kind of nursing care services do you like best? □

1=Good attitude-centered care service 2=Good nursing skill-centered care service 3=Good environment-centered nursing care service 4=Good health education guidance-centered care service

Note: If you are under 14 years of age, please ask your guardian to complete this form with you

**Basic information of the patient's department and the nurse in charge**

A1 Age years

A2 Gender 1=Male 2=Female □

A3 Education level □

1=Junior college or less 2=bachelor 3=Master 4=Doctor

A4 Nurse-staffing levels □

1=Registered nurse 2=Nurse practitioner 3=Supervisor nurse 4=Co-chief nurse

5= Chief nurse

A5 Years worked as a nurse □

1=1-10 2=11-20 3=21-30 4=31-40

B1 Which is the most important aspect of being a nurse? □

1= Keeping a good attitude 2=Good nursing technology 3= Keeping a good environment 4= Good health education guidance

Investigators_________ Investigation time _________

Auditor_________ Audit time _________
